# Twelve-Year Changes in Pre-Schoolers’ Oral Health and Parental Involvement in Children’s Dental Care: Results from Two Repeated Cross-Sectional Surveys in Lithuania

**DOI:** 10.3390/children11111380

**Published:** 2024-11-13

**Authors:** Apolinaras Zaborskis, Jaunė Razmienė, Augustė Razmaitė, Vilija Andruškevičienė, Julija Narbutaitė, Eglė Aida Bendoraitienė, Aistė Kavaliauskienė

**Affiliations:** 1 Department of Preventive Medicine & Health Research Institute, Faculty of Public Health, Medical Academy, Lithuanian University of Health Sciences, A.Mickevičiaus 9, LT-44307 Kaunas, Lithuania; 2 Department of Oral Health and Paediatric Dentistry, Medical Academy, Lithuanian University of Health Sciences, A.Mickevičiaus 9, LT-44307 Kaunas, Lithuania; jaune.razmiene@lsmu.lt (J.R.); augurazm0210@lsmu.lt (A.R.); vilija.andruskeviciene@lsmu.lt (V.A.); julija.narbutaite@lsmu.lt (J.N.); egleaida.bendoraitiene@lsmu.lt (E.A.B.); 3 Department of Orthodontics, Faculty of Odontology, Medical Academy, Lithuanian University of Health Sciences, A. Mickevičiaus 9, LT-44307 Kaunas, Lithuania; aiste.kavaliauskiene@lsmu.lt

**Keywords:** children, parents, oral health, early childhood caries, oral health-related behaviour, parental attitudes, repeated cross-sectional surveys, temporal trends

## Abstract

Background and objectives: The role of parents and the family in promoting children’s oral health is increasingly acknowledged in the dental literature. This study aimed to investigate twelve-year changes in pre-schoolers’ oral health and parental involvement in children’s dental care using data from two repeated cross-sectional surveys. The objectives were (1) to assess the temporal changes in children’s dental health status and oral health-related behaviours, (2) to examine temporal changes in parental attitudes toward their children’s oral health care, and (3) to analyze the associations between observed changes. Materials and methods: Two identical cross-sectional surveys were conducted in 2010/2011 (*n* = 294) and 2023 (*n* = 304). In both surveys, parents answered questions regarding their children’s oral health care and completed the 38-item ‘Parental Attitudes toward Children’s Oral Health’ (PACOH) scale, while the dental health of their 3–7-year-old children was clinically assessed. Changes in the examined characteristics were analyzed using general linear models. Results: Significant positive changes (*p* < 0.001) were observed when comparing the surveys: the dmf-t (decayed, missing, filled teeth) score for deciduous teeth decreased from 5.56 to 3.31; the Silness–Löe Plaque Index decreased from 1.45 to 1.15; the percentage of children brushing their teeth regularly increased from 33.0% to 55.3%; the percentage of parents assisting their child with tooth brushing increased from 19.2% to 85.1%; and regular visits to a children’s dentist increased from 36.4% to 85.1%. Moreover, the study revealed better parental attitudes toward their children’s oral health care, as reflected by a change in the total score of the PACOH scale from 112 to 122. Conclusions: It was concluded that changes in parental attitudes played a crucial role in driving positive trends in oral health-related behaviours, which likely contributed to the improvement of dental health in children. Therefore, understanding and influencing parental attitudes can be essential for promoting good oral health skills and good oral health in young children.

## 1. Introduction

Numerous studies have emphasized the crucial role of parents and families in promoting children’s oral and general health [1,2,3]. In this regard, research has demonstrated a significant relationship between parents’ knowledge and attitudes and their children’s oral hygiene skills practice within the family setting [4], the control of sugar consumption between meals [5,6], ensuring regular dental visits for children [7,8,9], and other factors, the negative manifestations of which may contribute to the onset of dental caries in childhood [10,11]. Consequently, parents should be seen as a key social force in ensuring children’s prosperity in early life since they have the potential to improve the general oral health of the community’s next generation [12].

Early childhood, often referred to as the pre-school years, is indeed a critical period for children’s development across various domains [13]. For instance, routines like regular toothbrushing, the right skills of eating and choosing food, and consistent positive interactions with parents lay a strong foundation for future development. Establishing positive habits and behaviours at this stage can have a greater impact on their long-term outcomes than doing so later in life [10,14]. Therefore, comprehensive data on oral health and its predictors in early childhood help to properly plan dental caries prevention programmes for children and implement effective methods of preserving healthy teeth for a long time [15,16]. The prevention of early childhood caries (ECC) should be integrated into existing primary health care programmes, especially those for maternal and child health [17]. However, in Lithuania, as well as, it seems, globally [18,19], epidemiological research on the oral condition of pre-school-aged children has not been conducted as much as that on school-aged children in order to help properly plan dental caries prevention programmes. Tooth decay among pre-school children remains a concern in Lithuania [20,21], but we lack a comprehensive database of epidemiological studies to track changes in children’s dental health over time. It is unclear how parents contribute to the development of children’s oral hygiene skills, what their attitudes are toward maintaining their children’s oral health, whether these attitudes have changed in the past decade, and, finally, whether these changes could impact children’s oral health.

The relevance of answers to these questions is supported by studies that have examined the relationship between parents’ attitudes toward children’s oral health and their children’s oral health-related behaviour or overall oral health outcomes [3,11,22,23,24,25,26]. Various instruments have been developed and utilized in previous cross-sectional studies to measure the level of parents’ or caregivers’ knowledge, beliefs, and attitudes regarding pre-school children’s oral health and hygiene skills. The Parental Attitudes toward Children’s Oral Health (PACOH) scale/questionnaire developed by Pine and Adair et al. (2004) in a multicenter study is one of such attempts that received particular attention [27,28]. The authors state that the components of this instrument are grounded in psychological frameworks, including the Theory of Planned Behaviour [29], the Health Belief Model [30], and the Health Locus of Control model [31]. A structural analysis of the scale conducted in our previous study [32] revealed a strong relationship between positive parental attitudes toward their children’s oral health and oral health-promoting behaviours in children, such as regular tooth brushing, dental visits, and parental assistance with brushing. Consequently, it was hypothesized that a positive trend in parental attitudes would correlate with improvements in children’s dental health status and oral health-related behaviours. Repeated public health surveys, especially those conducted over long intervals, provide valuable information to health policymakers and health care providers about changes in population health, including oral health.

To the best of our knowledge, long-term changes in the oral health and oral health-related behaviour of Lithuanian pre-school-aged children have not yet been analyzed. The opportunity for such an analysis arose after conducting two repeated surveys in the same region, using the same research methodology in 2010/2011 and 2023. Both studies also employed the PACOH questionnaire. Therefore, it was rational to conduct a long-term study using individual-level data to investigate temporal changes in the oral health of pre-school children, comparing them to changes in parental attitudes and practices. The results of such a study would enable us to assess the significance of changes in parents’ attitudes toward children’s oral health care for the benefit of their children.

To fill this gap, the current study aims to investigate changes in oral health over a period of 12 years (between 2010/2011 and 2023) in a sample of pre-school children in Lithuania, examining the temporal changes in parental attitudes toward their children’s oral health care. The objectives were (1) to assess the temporal changes in children’s dental health status and oral health-related behaviours, (2) to examine temporal changes in parental attitudes toward their children’s oral health care, and (3) to analyze the association between observed changes. Based on these objectives, the initial hypothesis was that the twelve-year changes in pre-schoolers’ oral health and oral health behaviour would be associated with improvements in their parents’ attitudes toward children’s oral health care.

## 2. Materials and Methods

### 2.1. Study Design, Participants and Ethical Consideration

An observational study design was used in this research. The data were derived from two identical cross-sectional surveys conducted in 2010/2011 and 2023. The target population was children of pre-school age and their parents living in the Kaunas region (Kaunas and surrounding rural area), Lithuania. No specific intervention was implemented during the study period in the selected population.

The sample size was estimated to ensure the reliability and validity of the results regarding children’s oral health. Assuming a 50% caries prevalence (the highest uncertainty), a minimum sample size of 610 participants (305 participants per survey) was calculated using G*Power 3.1 software (University of Dusseldorf, Dusseldorf, Germany) [33]. This calculation, based on a z-test, was designed to detect a 10% difference in caries prevalence between the two surveys, with a significance level of α = 0.05 and a power of 0.8.

Pre-schoolers were recruited from a list of nurseries in the Kaunas region, which included approximately 40 government nurseries at the time of the surveys. From this list, six nurseries were randomly selected—three from Kaunas and three from the surrounding rural areas. The number of children attending these nurseries met the required sample size for both surveys. The inclusion criteria for participation in the present study were parental consent for the child’s examination and willingness to participate in the questionnaire survey, as well as the child’s willingness to participate in oral examination. No specific exclusion criteria for children’s health were applied.

In the first survey, data were collected over 12 months, from November 2010 to October 2011. Anticipating a 30% non-response rate, 386 parents were invited to participate. Of these, 312 parents took part, resulting in a response rate of 81%. However, data analysis included only 294 parent/child pairs, as both the parents’ questionnaire and the child’s clinical oral examination were completed. The second survey was conducted from April to June 2023. Out of 425 parents invited, 307 participated, yielding a response rate of 71%. After data cleaning, 302 parent/child pairs were included in the final analysis.

The study received ethical approval from the Kaunas (Lithuania) Regional Committee for Biomedical Research Ethics (No. BE-2-19 for the survey in 2010/2011 and No. 2023-BE-10-0003 for the survey in 2023). Written informed consent was obtained from all parents of the participating children, as well as from the leading persons of all nurseries visited.

### 2.2. Questionnaire

The self-administered anonymous questionnaire was completed by either the father or the mother.

The first section of the questionnaire gathered information about the respondent’s sex (father or mother), age, education level (whether they graduated from university or college), household location (city or rural area), as well as the child’s sex and age. Respondents were also asked about their child’s dental health, toothbrushing habits, and visits to dentist. The child’s dental health was assessed based on the parents’ responses to the following question: ‘How do you assess the child’s dental health?’ Responses of ‘teeth are healthy, no treatment required’ and ‘the teeth are healed’ were categorized as ‘healthy teeth’, while responses of ‘treatment is required’ and ‘I don’t know the condition of the child’s teeth’ were categorized as ‘not healthy teeth or undefined status’. The frequency of toothbrushing was recorded as follows: 1 = ‘more than once a day’, 2 = ‘once a day’, 3 = ‘at least once a week, but not daily’, 4 = ‘less often than once a week’, and 5 = ‘never’. Response options were classified as either regular toothbrushing (response option: ‘more than once a day’) or irregular toothbrushing (the remaining options). The frequency of dental visits was classified as regular (response options: ‘two times or more a year’ and ‘at least once a year’) or irregular (response options: ‘irregularly’ and ‘never’).

The second section of the questionnaire included the 38-item PACOH scale, adapted from the study by Adair et al. (2004) [27,28]. In accordance with this study, the statements were categorized into three dimensions. The first dimension consisted of 14 items reflecting parental attitudes toward their children’s toothbrushing, including the importance and intention of brushing their children’s teeth. The second dimension comprised 9 items related to parental attitudes toward their children’s sugar snacking, including the importance and intention of controlling sugar intake. The third dimension included 15 items addressing parental attitudes toward the prevention of children’s dental decay, focusing on the perceived seriousness of tooth decay in children. In this paper, we refer to these three dimensions as TB, SS, and DD.

Responses to the PACOH scale items were rated on a 4-point Likert scale, with the following item scores: 1 = ‘strongly agree’, 2 = ‘agree’, 3 = ‘disagree’, and 4 = ‘strongly disagree’. If none of these response categories were selected, the item was assigned a value of 2.5. For statements where agreement indicated a positive attitude towards the child’s dental health, the response values were inverted. The scores for each PACOH dimension were then summed, with a higher sum score indicating a more positive attitude from the respondent. Both numerical continuous PACOH sum score values and median dichotomized values (<120 scores and ≥120 scores) were used in the analysis.

We used the Lithuanian version of the PACOH scale, which had been translated from English, validated, and applied in previous studies [11,21,32]. The internal reliability values (Cronbach’s alpha) for the scale were 0.794 for the 2010/2011 survey and 0.790 for the 2023 survey.

### 2.3. Clinical Examination

Clinical oral examinations were conducted in the nurseries under standardized conditions, utilizing a portable dental unit along with convertible chairs for both patients and examiners. The dental unit featured a fibre optic light source, compressed air, and a suction device. Dental status assessments were carried out using a standard explorer and a dental mirror. All examinations were performed by dentists who were trained prior and standardized. The researchers assessed the caries experience and prevalence in deciduous teeth, as well as children’s oral hygiene.

Dental caries experience was assessed using the dmf-t index, which reflects the total number of decayed (d), missing (m), and filled (f) deciduous teeth (WHO Oral health surveys basic methods 5th ed. [34]). Caries prevalence was determined by calculating the proportion of individuals with a dmf-t score greater than 0, relative to the total number of individuals examined. 

Oral hygiene status was evaluated using the Silness–Löe Plaque Index, which measures the amount and location of plaque. A probe is run along the surface of a tooth, with results categorized as follows: 0 = no plaque; 1 = plaque located on the gums and tooth neck area; 2 = plaque visible on the tooth neck area and interdentally; 3 = plaque covering the entire surface of the tooth. The plaque index is calculated by summing the scores and dividing by the number of teeth. The index is scored as follows: 0—excellent oral hygiene; 0.1 to 0.9—good; 1.0 to 1.9—satisfactory; and 2.0 to 3.0—poor [35].

### 2.4. Data Analysis

Data were analyzed using the IBM SPSS Statistics software (version 21; IBM SPSS Inc., Chicago, IL, USA). The analysis was conducted using both datasets for each survey and a merged dataset, which included the time (survey number) component as a separate variable. Descriptive statistics were employed to summarize the characteristics of the sample. Respondents’ responses were estimated by frequencies (*n*) and percentages (%). To describe continuous numeric measures (e.g., age), we used means and standard deviations as well as median-based dichotomized values. A Pearson’s correlation test was utilized to examine the relationship between observed variables. A bivariate analysis was carried out to identify differences between surveys. The Chi-square and z-tests were used to compare percentages of categorical variables (oral health status, oral hygiene status, toothbrushing frequency, etc.), while the t-test was utilized to assess differences in the means of continuous variables (sum score of the PACOH scale, dmf-t index, etc.). A *p*-value ≤ 0.05 was considered to indicate statistically significant differences, and the confidence interval (CI) was set at 95%.

A general linear model (GLM) analysis was utilized to calculate marginal means with a 95% CI for continuous variables, adjusting for socio-demographic variables. The linear regression approach of GLM was used to analyze the time trend of parental attitudes (sum score of PACOH scale) and their relationship with socio-demographic factors. Additionally, a GLM analysis was conducted to assess the impact of the 12-year period between surveys on changes in oral health outcomes and to examine their association with socio-demographic factors, parental attitudes, and oral health-related behaviours. Several models were tested: the first included only the time component, while subsequent models varied by the number of predictors and the sequence in which they were included. All predictors were dichotomized to compare their regression weights (B). Partial eta squared (η^2^) was used to estimate effect sizes of each predictor [36]. A hypothesis that multicollinearity between predictors might exist was tested and rejected. No significant interactions between predictors was identified.

## 3. Results

### 3.1. Socio-Demographic Characteristics

Table 1 compares socio-demographic characteristics of respondents and their children between surveys in 2010/2011 and 2023. It can be noticed that in the second survey, the respondents were older and had a higher level of education, and a greater proportion came from rural areas compared to the first survey. Meanwhile, there were no significant differences between the surveys in terms of the respondents’ sex and the sex and age of the children.

### 3.2. Change in Parental Attitudes Towards Children’s Oral Health

Table 2 compares parental attitudes towards children’s oral health between the 2010/2011 and 2023 surveys using the PACOH scale. The results from the second survey showed a significant increase in the sum scores for all dimensions, as well as the total PACOH scale, compared to the first survey.

Table 3 depicts the estimated regression weights used to predict the sum scores of the total PACOH scale and its dimensions, based on survey year and socio-demographic variables. The data indicate that the time factor (survey year), as also shown in Table 2, had a particularly significant positive effect on all dimensions of parental attitudes toward their child’s oral health. In contrast, the impact of socio-demographic factors was smaller and did not significantly affect all dimensions. Specifically, older parents had a lower assessment of the importance of their child’s need for tooth brushing (dimension TB, B = −1.29, *p* < 0.001), while parents with higher education had a more positive assessment of the need for dental caries prevention (dimension DD, B = 1.76, *p* < 0.001). Additionally, parents from rural areas showed lower levels of positive attitudes across all dimensions compared to those from urban areas.

### 3.3. Change in Children’s Oral Health and Oral Health-Related Behaviour

Table 4 compares children’s oral health and oral health-related behaviour between the 2010/2011 and 2023 surveys using clinical and questionnaire measures. All these results show an excellent improvement in children’s oral health and their related behaviour over the 12-year period. A dmf-t index significantly decreased in 2.28 (95% CI: 1.66–2.90) units. This change was accompanied by a significant decrease in 31.1% (95% CI 24.2–38.0) of the caries prevalence. It can be assumed that with a significant (1.68 times) increase in the number of children who brush their teeth regularly, the number of children whose oral hygiene was rated excellent also increased (from 14.3% to 33.3%). The increased concern of parents for the health of their children’s teeth is shown by their more frequent provision of help brushing their children’s teeth (2.76 times) and regular visits to their children’s dentist (2.34 times).

### 3.4. Association Between Parental Attitudes and Children’s Oral Health over Time

To verify the reality of this association, we analyzed several general linear models, which differed in the number of predictors and the sequence of their inclusion in the model.

First, we analyzed the association between the dmf-t index and the sum score of the total PACOH scale. The correlation between these variables was inverse in both the first survey (*r* = –0.092, *p* = 0.117) and the second survey (*r* = –0.243, *p* < 0.001), indicating that higher PACOH scores were associated with lower dmf-t index values.

Table 5 presents the results of the analysis examining the association between the dmf-t index and the sum score of PACOH scale, considering the 12-year interval between surveys and other predictors. The time component emerged as a significant predictor, influencing changes in the dmf-t index value regardless of the number of predictors included in the model. When parental attitudes toward children’s oral health, represented by the PACOH sum score (Model 2), were added to the list of predictors, they showed a positive effect size almost equal to that of the time component. The influence of parental attitudes remained significant even after adjusting for socio-demographic factors (Model 3). However, the significance and effect size of the parental attitudes predictor diminished when oral health-related behavioural factors were included in the model (Models 4 and 5). Regular toothbrushing and parental assistance with brushing showed particularly strong effects in predicting caries experience. The complete set of predictors explained more than a quarter of the total variation in dmf-t index values (adjusted R^2^ = 26.4%).

We then analyzed the changes in the frequency of children’s toothbrushing. A higher frequency of tooth brushing was correlated with a lower Silness–Löe plaque index (*r* = 0.689, *p* < 0.001 in the first survey; *r* = 0.316, *p* < 0.001 in the second survey) and a lower dmf-t index (*r* = 0.467, *p* < 0.001 in the first survey; *r* = 0.276, *p* < 0.001 in the second survey). However, a significant correlation between a higher frequency of children’s toothbrushing and a higher total score on the PACOH scale was observed only in the second survey (*r* = 0.253, *p* < 0.001). Table 6 presents the results of the analysis of the association between the frequency of regular children’s toothbrushing and the total PACOH scale score, considering the 12-year interval between surveys. The time component had a significant effect on changes in regular toothbrushing habits. The total PACOH scale score, which reflects parental attitudes toward children’s oral health, emerged as a significant predictor of regular toothbrushing frequency in children.

## 4. Discussion

The present study is unique, as it is the first oral health time-trend study in pre-school children with a focus on parental attitudes and practices towards children’s oral health care. A comparison of data from two surveys revealed significant improvements in the oral health indicators of pre-school children over a recent 12-year period. The prevalence of caries, the dmf-t index, and the Silness–Löe plaque index decreased, and the percentage of children who regularly brushed their teeth increased. Additionally, there was a notable increase in parental involvement: attitudes toward children’s oral health care improved, more parents began helping their children brush their teeth, and the number of parents regularly taking their children to the dentist also rose. These findings prompted the testing of the hypothesis that positive changes in parental attitudes could positively influence children’s oral health. The results of the further data analysis provided strong support for acceptance of this hypothesis.

Testing this hypothesis based solely on repeated cross-sectional surveys, without prospective research data, presents significant challenges. To address this, no alterations were made in the sampling process, methods, definitions, or criteria, ensuring consistency when comparing trends and relationships between variables over time. Next, the analysis was conducted using a merged dataset, which included the time component as a separate variable. This approach is particularly powerful for studies that seek to understand both cross-sectional and longitudinal relationships [37,38]. It offers several advantages. First, including time as a variable allows the analysis to account for trends or changes over time, making it possible to observe how the relationships between variables evolve. Second, by combining data from multiple surveys, the sample size increases, which can improve the robustness and reliability of the analysis, reducing the chances of random error or bias. Third, this approach allows one to directly compare results from different survey periods, helping identify differences or trends across time and making it easier to assess changes in the population being studied, and so on.

Dental caries remains one of the most prevalent childhood diseases worldwide, affecting a significant number of pre-school children [18,19,39]. However, during the last three decades, the global average prevalence of caries of deciduous teeth decreased slightly by 3%, with the largest decreases in high-income countries [39]. In most industrialized countries in northern Europe, in North America, and in Australia and New Zealand, the prevalence of dental caries is decreasing, often linked to an increasing use of fluorides, to various types of dental health education programmes, etc. [17]. In Scandinavia, where all pre-school children are included in an organized dental care programme, dental caries has been decreasing markedly since the 1970s and the beginning of the 1980s, while more and more children are totally free of the disease at present [40,41].

In Lithuania, a comprehensive nationwide epidemiological study conducted in 2010–2012 revealed that the prevalence and severity of tooth decay among 4–6-year-old children were high, with a prevalence rate of 89.7% and an average dmf-t (decayed, missing, filled teeth) score of 7.9 (SD = 4.94) [21]. Unfortunately, data on the long-term trends of dental caries in children remain scarce. Only one comprehensive epidemiological study on pre-school children’s oral health, conducted in Kaunas (a city in Lithuania), stands out as particularly noteworthy. The results of this three-year preventive programme demonstrated that systematic oral hygiene education and specific caries prevention measures—such as fluoride varnish and gel applications, along with toothbrushing with fluoride toothpaste—are effective in reducing both the prevalence and severity of caries in deciduous teeth [42]. However, in practice, Lithuanian pre-schoolers still lack access to long-term prevention programmes. According to Lithuanian professional dentists [21], dental care for pre-schoolers is often limited to symptomatic treatment, with preventive programmes conducted only sporadically. These programmes do not cover the entire child population, and there is no ongoing registration of data on the temporal trends of dental caries in children.

Given the previously unfavourable dental health prognosis for Lithuanian children, the significant improvement in the dental health of pre-schoolers over the past 12 years observed in our study is truly surprising. We hypothesize that this improvement is due to a shift in parents’ attitudes toward the importance of their children’s oral health, as well as more parental involvement in ensuring the regularity of their children’s tooth brushing, assisting their children with brushing, and ensuring regular visits to the dentist. Because no specific intervention in parents was conducted, the positive shift in parental attitudes and higher parents’ involvement in children’s oral health care can likely be attributed to the overall rise in health literacy within the population, driven by the influence of the internet, radio, television, and printed materials [43]. Additionally, expanded communication among parents, facilitated by social networks, has also played a positive role in this process [44].

A significant positive change in parental attitudes was observed across all dimensions (TB, SS, and DD) during the study period. However, none of these dimensions were entirely independent of factors such as the parent’s and child’s sex or the child’s age. Notably, only parental attitudes toward toothbrushing (dimension RB) were negatively influenced by older parental age. Respondents with higher education levels exhibited more positive attitudes specifically towards preventing dental caries (dimension DD), while urban respondents displayed more positive attitudes across all dimensions of their children’s oral health care compared to rural respondents. These findings align with those of previous studies, which suggest that individuals’ attitudes are influenced by education level and urban or rural residence. These factors may directly affect attitudes or exert indirect influence through variables like occupation, income, access to information, health care infrastructure, and social networks [8,45].

The findings of our study are in line with the theoretical model developed from the Theory of Planed Behaviour [29], which provides evidence that changing the attitudes of parents can change not only the behaviour of the parents, but also the behaviour of the children. Pre-school children spend most of their time with their parents, so family-based oral health care is important for the further development of the child’s skills [25,46]. Teaching good oral hygiene begins at home, where parents teach their children oral hygiene routines by being involved in the teaching process themselves and by setting an example of personal skills for their offspring. Thus, during early childhood, parental supervision and helping to develop any child’s skills are the most common and effective measures to ensure a child’s regular tooth brushing [25,47], which is a very important factor that ensures good oral hygiene, dental plaque removal, and dental decay prevention [48,49,50]. Hence, the role of parents and family as moderators of children’s oral health and their related behaviours is increasingly acknowledged in the dental literature [3,12].

Our study confirms that children of parents with higher education tend to have healthier teeth, consistent with findings from other studies [8,51,52]. Among families with children in the early mixed dentition stage, parents with higher education levels are more likely to possess better oral health knowledge and to seek preventive care measures, such as pit and fissure sealants [8,53]. Additionally, in these families, children’s oral hygiene habits are more likely to align with dental recommendations, including brushing teeth twice a day or more frequently [53]. The likelihood of pre-school children visiting a dentist also increases with the parents’ level of education [7,8,9]. Research in Spain and Italy further supports the concept that parents with higher education are more knowledgeable about oral health factors [45,53]. In the second survey of our study, a higher percentage of respondents had higher education compared to the first survey. Furthermore, our study showed that parents with higher education had more positive attitudes towards their child’s oral health care, which corresponds to the observations of other authors [8,45]. Therefore, considering the findings from the studies mentioned above, it is likely that the higher education level among respondents in the second survey positively influenced the oral health outcomes observed in their children.

We found that parental attitudes toward their child’s oral health were significantly associated with the dmf-t index, with higher PACOH scale scores related to lower dmf-t scores. The influence of parental attitudes had a positive effect size nearly equal to that of the time component and remained significant even after adjusting for socio-demographic factors. These findings align with previous research, which highlights the crucial role of parental attitudes in preventing caries in pre-school children [54]. Based on this, it can be concluded that during early childhood, parental attitudes toward supervising and developing tooth brushing skills, as well as controlling sugar consumption, are among the most effective preventive measures against caries [22,23,24,25]. Further analysis is needed to explain why the direct impact of parental attitudes on dental health becomes less significant when factors such as regular toothbrushing and parental assistance with children’s tooth brushing are included in the model. However, our results indicate that these factors are closely related to parental attitudes, suggesting that they may act as mediators in the association between parental attitudes and children’s dental health. Consequently, the effect of parental attitudes on dental health may be indirect, operating through these mediators. Verification of this assumption requires more detailed investigation.

In addition to the previously mentioned limitation, this study has several other limitations worth noting. First, the research was conducted in a specific region of Lithuania. Given that the analyzed indicators may vary significantly across different regions, the findings have limited generalizability to the entire country. Second, our study did not address a child’s dietary habits, specifically sugar consumption, so we could not compare parental attitudes between groups of children based on the frequency of sugar consumption. Previous research [11,27] suggests that temporal trends in children’s sugar consumption may be associated with changes in the second (SS) dimension, which pertains to parental attitudes toward their child’s sugar snacking, including the importance and intention of controlling sugar intake. Third, there were differences in the socio-demographic characteristics of respondents between the 2010/2011 and 2023 surveys. In the second survey, respondents were older and had higher levels of education, and a greater proportion were from rural areas compared to the first survey. As discussed earlier, these characteristics may influence parental attitudes and children’s oral health. However, due to their varying directions of influence, the overall cumulative effect may not be significant. Fourth, our study did not aim to identify strategies for improving parental attitudes or preventing dental decay in children. Combining research on attitudes and habits could clarify the timing and methods for implementing attitude-focused interventions that are most effective in strategies for changing oral health-related behaviours [54,55,56]. Finally, the cross-sectional design of this study, despite being supported by repeated surveys, limits our ability to draw causal inferences. Longitudinal studies would be valuable in understanding the dynamics of the relationship between parental attitudes and children’s oral health over time and in identifying causality in this relationship. 

Despite the aforementioned limitations, this study has several noteworthy strengths. It highlights the crucial role of parents’ attitudes in shaping children’s oral health-related behaviour and, consequently, in improving oral health outcomes. This finding not only contributes to the existing body of knowledge but also calls for further research. Future studies are warranted to delve into the dynamics of relationships between parental attitudes toward oral health; oral health-related behaviour, including dietary habits; and oral health outcomes in children. Additionally, research focusing on interventions targeting parental attitudes is recommended. Based on the findings of this study, it is reasonable to assume that such interventions could enhance parents’ awareness of the importance of developing children’s oral hygiene skills and promoting good oral health. 

## 5. Conclusions

Significant positive changes in pre-schoolers’ oral health outcomes and parental attitudes toward their children’s oral health care were observed in a 2023 survey, compared to an identical survey from 2010/2011. The study highlighted that changes in parental attitudes played a crucial role in driving improvements in oral health-related behaviours, which likely contributed to better dental health in children. Therefore, understanding and influencing parental attitudes can be essential for promoting good oral health skills and good oral health in young children.

## Figures and Tables

**Table 1:** Socio-demographic characteristics of respondents and their children.

Characteristic	Survey in 2010/2011 (*n* = 294)		Survey in 2023 (*n* = 302)		*p*-Value
	*n*/Mean	%/SD	*n*/Mean	%/SD	
Sex of respondents:					
Males (fathers)	46	15.6	40	13.2	0.404
Females (mothers)	248	84.4	262	86.8	
Age of respondents:					
Mean (SD)	33.4	5.39	35.3	4.25	<0.001
<35 years	197	67.0	164	54.3	0.002
≥35 years	97	33.0	138	45.7	
Education level of respondents:					
Less than college or university	96	32.8	76	25.2	0.041
College or university	197	67.2	226	74.8	
Nursery location:					
Urban area	220	74.8	202	66.9	0.033
Rural area	74	25.2	100	33.1	
Sex of children:					
Boys	152	51.7	164	54.3	0.524
Girls	142	48.3	138	45.7	
Age of children:					
Mean (SD)	5.02	0.82	5.18	0.84	
<5 years	134	45.6	151	50.0	0.280
≥5 years	160	54.4	151	50.0	

**Table 2:** Comparison of parents’ attitudes towards children’s oral health between surveys.

Sum Score	Survey in 2010/201 (*n* = 294)		Survey in 2023 (*n* = 302)		Increase		*p*-Value
	Mean	SD	Mean	SD	Mean	95% CI	
Dimension TB ^1^	44.34	4.93	47.85	4.43	3.51	2.76–4.27	<0.001
Dimension TB ^2^	43.78	6.05	47.52	6.10	3.75	2.99–4.50	<0.001
Dimension SS ^1^	25.63	4.64	28.31	4.00	2.68	1.98–3.37	<0.001
Dimension SS ^2^	25.27	5.68	27.99	5.72	2.72	2.01–3.43	<0.001
Dimension DD ^1^	43.36	4.85	46.53	5.09	3.17	2.37–3.97	<0.001
Dimension DD ^2^	42.94	6.38	46.12	6.43	3.18	2.39–3.98	<0.001
Total PACOH scale ^1^	113.33	10.24	122.69	10.00	9.36	7.73–10.99	<0.001
Total PACOH scale ^2^	111.99	13.01	121.64	13.12	9.65	8.02–11.27	<0.001

Notes: TB: parental attitudes toward their children’s toothbrushing; SS: parental attitudes toward their children’s sugar snacking; DD: parental attitudes toward prevention of children’s dental decay; PACOH: parental attitudes toward children’s oral health. ^1^ crude values; ^2^ values adjusted for socio-demographic variables.

**Table 3:** Estimates of regression weights to predict sum scores of total PACOH scale and its dimensions.

Characteristic	Regression Weights (B-Values)			
	Dimension TB	Dimension SS	Dimension DD	Total PACOH
Year of the survey:				
2010/2011 ^#^	0	0	0	0
2023	3.75 ***	2.72 ***	3.18 ***	9.65 ***
Sex of respondents:				
Males (fathers) ^#^	0	0	0	0
Females (mothers)	–0.10	0.53	–0.80	–0.37
Age of respondents:				
<35 years ^#^	0	0	0	0
≥35 years	–1.29 ***	0.15	–0.46	–1.60
Education level of respondents:				
Less than college or university ^#^	0	0	0	0
College or university	0.24	0.04	1.76 ***	2.04 *
Nursery location:				
Urban area ^#^	0	0	0	0
Rural area	–1.35 **	–0.88 *	–1.31 **	–3.54 ***
Sex of children:				
Boys ^#^	0	0	0	0
Girls	–0.45	0.70	–0.45	–0.19
Age of children:				
<5 years ^#^	0	0	0	0
≥5 years	–0.59	–0.38	–0.54	–1.52

Notes: TB: parental attitudes toward their children’s toothbrushing; SS: parental attitudes toward their children’s sugar snacking; DD: parental attitudes toward prevention of children’s dental decay; PACOH: parental attitudes toward children’s oral health care. ^#^ reference category; * *p* < 0.05; ** *p* < 0.01; *** *p* < 0.001.

**Table 4:** Comparison of children’s oral health and oral health-related behaviour between surveys.

Characteristic	Survey in 2010/2011 (*n* = 294)		Survey in 2023 (*n* = 302)		*p*-Value
	*n*/Mean	%/SD	*n*/Mean	%/SD	
Parental assessment of children’s dental health:					
Healthy teeth	164	55.8	240	79.5	<0.001
Not healthy teeth or undefined status	130	44.2	62	20.5	
Dmf-t index:					
Mean (SD) ^1^	5.39	4.05	3.11	3.62	<0.001
Mean (SD) ^2^	5.56	4.90	3.31	4.92	<0.001
Caries prevalence	253	86.1	166	55.0	<0.001
Assessment of oral hygiene by Silness–Löe plaque index:					
Mean (SD) ^1^	1.45	0.86	1.15	1.02	<0.001
Mean (SD) ^2^	1.63	1.18	1.33	1.22	<0.001
excellent oral hygiene	42	14.3	95	33.3	<0.001
good	108	36.7	87	30.5	
satisfactory	114	28.8	68	23.9	
poor	30	10.2	35	12.3	
Child brushes their teeth:					
Regularly	97	33.0	167	55.3	<0.001
Irregularly	197	67.0	135	44.7	
Parents help their children brush their teeth:					
No	236	80.8	142	47.0	<0.001
Yes	56	19.2	160	53.0	
Visits to children’s dentist:					
Regular	107	36.4	257	85.1	<0.001
Irregular	187	63.6	45	14.9	

Notes: ^1^ crude values; ^2^ values adjusted for socio-demographic variables.

**Table 5:** Estimated regression weights (B) and partial η^2^ for predicting the dmf-t index value among pre-schoolers using general linear models.

Intercept and Predictors	Model 1		Model 2		Model 3		Model 4		Model 5	
	B	Partial η^2^	B	Partial η^2^	B	Partial η^2^	B	Partial η^2^	B	Partial η^2^
Intercept	5.39 ***	0.251	5.73 ***	0.152	5.70 ***		4.61 ***	0.067	4.64 ***	0.037
Year of the survey:										
2010/2011 ^#^	0		0		0		0		0	
2023	–2.28 ***	0.081	–1.93 ***	0.054	–1.94 ***	0.055	–0.96 **	0.012	–1.00 **	0.013
Sum score PACOH:										
<120 ^#^			0		0		0			
≥120			–1.21 ***	0.022	–1.00 **	0.015	–0.51	0.005	–0.44	0.003
Child brushes their teeth:										
Regularly ^#^							0		0	
Irregularly							2.10 ***	0.077	2.14 ***	0.081
Parents help the children brush their teeth:										
No ^#^							0		0	
Yes							–2.21 ***	0.071	–1.92 ***	0.053
Visits to children’s dentist:										
Regular ^#^							0		0	
Irregular							–0.14	0.000	–0.17	0.000
Sex of respondents:										
Males (fathers) ^#^					0				0	
Females (mothers)					0.58	0.003			0.56	0.003
Age of respondents:										
<35 years ^#^					0				0	
≥35 years					0.74	0.009			0.48	0.004
Education of respondents:										
Less than college or university ^#^					0				0	
College or university					–1.45 ***	0.027			–1.20 ***	0.021
Nursery location:										
Urban area ^#^					0				0	
Rural area					–0.11	0.000			–0.48	0.003
Sex of children:										
Boys ^#^					0				0	
Girls					–0.45	0.004			–0.36	0.003
Age of children:										
<5 years ^#^					0				0	
≥5 years					0.82 **	0.012			0.67 *	0.009
Adjusted R^2^ (%)		8.0		10.3	14.6			24.0		26.4

Notes: PACOH: parental attitudes toward their children’s oral health care; ^#^ reference category; * *p* < 0.05; ** *p* < 0.01; *** *p* < 0.001.

**Table 6:** Estimated regression weights (B) and partial η^2^ for predicting the regular toothbrushing among pre-schoolers using general linear models.

Characteristic	Model 1		Model 2		Model 3	
	B	Partial η^2^	B	Partial η^2^	B	Partial η^2^
Intercept	33.0 ***	0.398	29.1 ***	0.386	33.7 ***	0.253
Year of the survey:						
2010/2011 ^#^	0		0		0	
2023	22.3 ***	0.050	19.3 ***	0.034	21.1 ***	0.039
Sum score PACOH:						
<120 ^#^			0			
≥120			13.2 ***	0.016	12.8 **	0.015
Sex of respondents:						
Males (fathers) ^#^					0	
Females (mothers)					−0.3	0.000
Age of respondents:						
<35 years ^#^					0	
≥35 years					−6.3	0.004
Education level of respondents:						
Less than college or university ^#^					0	
College or university					0.3	0.000
Nursery location:						
Urban area ^#^					0	
Rural area					−5.2	0.002
Sex of children:						
Boys ^#^					0	
Girls					6.7	0.005
Age of children:						
<5 years ^#^					0	
≥5 years					4.2	0.002
Adjusted R^2^ (%)		4.9		7.0		7.2

Notes: PACOH: parental attitudes toward their children’s oral health care; ^#^ reference category; ** *p* < 0.01; *** *p* < 0.001.

## Data Availability

Dataset available on request from the corresponding author.
